# Different Sensitivity of Flower-Visiting Diptera to a Neonicotinoid Insecticide: Expanding the Base for a Multiple-Species Risk Assessment Approach

**DOI:** 10.3390/insects15050317

**Published:** 2024-04-29

**Authors:** Cátia Ariana Henriques Martins, Celeste Azpiazu, Jordi Bosch, Giovanni Burgio, Maria Luisa Dindo, Santolo Francati, Daniele Sommaggio, Fabio Sgolastra

**Affiliations:** 1 Dipartimento di Scienze e Tecnologie Agro-Alimentari, Alma Mater Studiorum Università di Bologna, 40127 Bologna, Italy; catia.martins2@unibo.it (C.A.H.M.); giovanni.burgio@unibo.it (G.B.); marialuisa.dindo@unibo.it (M.L.D.); santolo.francati2@unibo.it (S.F.); 2 CREAF, Centre de Recerca Ecològica i Aplicacions Forestals, Universitat Autònoma de Barcelona, 08193 Bellaterra, Spain; c.azpiazu@creaf.uab.cat (C.A.); jordi.bosch@uab.cat (J.B.); 3 Universidad Politécnica de Madrid, 28040 Madrid, Spain; 4 Dipartimento di Scienze della Vita, Università di Modena e Reggio Emilia, 41121 Modena, Italy; daniele.sommaggio@unimore.it; 5 National Biodiversity Future Center (NBFC), Piazza Marina 61, 90133 Palermo, Italy

**Keywords:** pollinators, Diptera, species sensitivity distribution, neonicotinoid pesticide, fecundity

## Abstract

**Simple Summary:**

Insect pollinators play an essential service in agricultural systems, but are commonly exposed to pesticides. Although pollinators are present in several insect orders, above all dipterans, information on pesticide sensitivity is mostly restricted to bees. We assessed the sensitivity of two hoverflies (*Sphaerophoria rueppellii*, *Eristalinus aeneus*) and one tachinid fly (*Exorista larvarum*) to a neonicotinoid insecticide (Confidor^®^, imidacloprid). We adapted the standardized methodology of acute contact exposure in honey bees to build dose–response curves and calculate median lethal doses (LD_50_) for the three species. *S. rueppelli* was the most sensitive, *E. aeneus* the least. Results were compared with those available in the literature for other pollinator species using a species sensitivity distribution (SSD) approach: as a result, the 95th percentile of pollinator species would be protected by a safety factor of 100 times the *Apis mellifera* endpoint. Dipterans were less sensitive to imidacloprid than most bee species. We measured the number of eggs laid following exposure to different insecticide doses and assessed the potential trade-off between oviposition and survival through the sublethal sensitivity index (SSI). Pesticide exposure had a significant effect on fecundity, and SSI values indicated that oviposition is a sensitive endpoint for the three dipteran species tested.

**Abstract:**

Insects play an essential role as pollinators of wild flowers and crops. At the same time, pollinators in agricultural environments are commonly exposed to pesticides, compromising their survival and the provision of pollination services. Although pollinators include a wide range of species from several insect orders, information on pesticide sensitivity is mostly restricted to bees. In addition, the disparity of methodological procedures used for different insect groups hinders the comparison of toxicity data between bees and other pollinators. Dipterans are a highly diverse insect order that includes some important pollinators. Therefore, in this study, we assessed the sensitivity of two hoverflies (*Sphaerophoria rueppellii*, *Eristalinus aeneus*) and one tachinid fly (*Exorista larvarum*) to a neonicotinoid insecticide (Confidor^®^, imidacloprid) following a comparative approach. We adapted the standardized methodology of acute contact exposure in honey bees to build dose–response curves and calculate median lethal doses (LD_50_) for the three species. The methodology consisted in applying 1 µL of the test solution on the thorax of each insect. *Sphaerophoria rueppelli* was the most sensitive species (LD_50_ = 10.23 ng/insect), and *E. aeneus* (LD_50_ = 18,176 ng/insect) the least. We then compared our results with those available in the literature for other pollinator species using species sensitivity distribution (SSD). Based on the SSD curve, the 95th percentile of pollinator species would be protected by a safety factor of 100 times the *Apis mellifera* endpoint. Overall, dipterans were less sensitive to imidacloprid than most bee species. As opposed to most bee species, oviposition and fecundity of many dipteran species can be reliably assessed in the laboratory. We measured the number of eggs laid following exposure to different insecticide doses and assessed the potential trade-off between oviposition and survival through the sublethal sensitivity index (SSI). Exposure to imidacloprid had a significant effect on fecundity, and SSI values indicated that oviposition is a sensitive endpoint for the three dipteran species tested. Future studies should integrate this information related to population dynamics in simulation models for environmental risk assessment.

## 1. Introduction

Insect pollinators play an essential role in ecosystem functioning and crop pollination [1,2]. Although bees usually take most of the credit for this ecosystem service, awareness of the importance of other insect pollinators is growing [3]. Dipterans, in particular, are one of the most species-rich insect orders, comprising more than 160,000 species grouped in ca. 160 families, of which at least 75 are flower visitors [4,5,6]. Different groups of flies, including hoverflies (Syrphidae), bee flies (Bombyliidae) and many groups of muscoid flies, visit flowers mostly to feed on pollen and nectar, but also to find prey and hosts for their larvae, oviposit, mate and take shelter [7]. Several studies show that flies may be highly effective pollinators [3,8,9,10] and highlight their contribution to wild flower and crop pollination by visiting at least 555 plant species, including over 100 cultivated plants [3,11,12]. Excluding Hymenoptera, most of which are bees, Diptera is the most frequent flower-visiting order (72% of crops) [13]. Based on their dual role as pollinators [5,14,15] and biological control agents [16,17] some studies advocate for the use of fly populations in integrated pest management [10].

Declines in pollinator abundance and diversity over the last century have been mostly documented for bees [18,19] and to a lesser extent hoverflies [20,21,22]. Importantly, these declines are partly associated with agricultural intensification and its strong reliance on systemic and non-systemic pesticides, including seed treatments [23,24,25]. To safeguard the well-being and survival of pollinators and other non-target organisms, pesticides undergo a process of environmental risk assessment [26,27]. However, non-bee pollinators are poorly represented in current environmental risk assessment. Bee risk assessment schemes rely on a surrogate species, the western honey bee, *Apis mellifera* L., and are based on the calculation of the dose of pesticide that is lethal to 50% of the population (LD_50_) following acute exposure [28,29]. This approach does not account for potential sensitivity differences among bee species [30,31,32,33,34,35]. Recent studies have compared the sensitivity of honey bees and other bees, including bumblebees, mason bees and stingless bees [30,32,36,37,38]. Non-bee pollinators are also overlooked in risk assessment procedures to assess the effects of pesticides on non-target arthropods [39]. For these reasons, the European Food Safety Authority recently published a road map to expand environmental risk assessment to additional pollinator species [40]. In addition, the few studies addressing pesticide sensitivity in non-bee pollinator taxa use different methodological procedures, hindering direct comparison with results obtained on bees [41,42,43].

In this study, we assessed the toxicity of a neonicotinoid insecticide (imidacloprid) in two hoverflies, *Sphaerophoria rueppellii* (Wiedemann) (Diptera: Syrphidae) and *Eristalinus aeneus* (Scopoli) (Diptera: Syrphidae), and a parasitoid fly, *Exorista larvarum* (L.) (Diptera: Tachinidae). The three tested species play important roles as pollinators for several plant species and in various ecological contexts. *Exorista larvarum* is a polyphagous gregarious larval parasitoid of several Lepidoptera, such as the gypsy and cabbage moths, which are pests in forest and agricultural environments, respectively [44]. It is also a common visitor of Apiaceae [45]. *Sphaerophoria rueppellii* is an aphidophagous hoverfly [46,47]. Populations of this species are commercially available due to their high effectiveness as biological control agents of pest aphids and as a pollinator of some crops [48]. *Eristalinus aeneus* is a saprophagous hoverfly. The adults are effective pollinators of hybrid seed crops [49].

Due to their foraging activities in crop plants during adult stage, all these species can be routinely exposed to pesticides. Imidacloprid is a neurotoxic insecticide with a high affinity for the nicotinic acetylcholine receptor (nAChR). Due to its physicochemical characteristics, imidacloprid can easily translocate to all plant tissues, thus providing protection from a wide range of herbivorous (mainly sap-feeding) insects. Imidacloprid has been registered in 120 countries for use on over 140 crops against several insect pests, including aphids, whiteflies, and the Colorado potato beetle [50]. It was introduced in the market in 1991 and quickly became the most worldwide used insecticide, accounting for 41.5% of the neonicotinoid market in 2009 [50]. The widespread use of this compound, together with its systemic properties and long persistence in the environment, raised concern about its impacts on biodiversity, ecosystem functioning and services [51,52,53]. Due to its high acute toxicity to bees, its use in the EU was banned by the European Commission in 2018 [54]. Despite this ban, many EU Member States provide emergency authorizations for its use, and it is still widely used in many parts of the world [55].

Historically, pesticide acute toxicity to honey bees has been tested following the protocols developed by the Organisation for Economic Cooperation and Development (OECD). These protocols are currently used for honey bees and, with some minor adaptations, for bumblebees [56,57,58,59]. However, similar standard protocols for non-bee pollinators are lacking. To cover this gap and gain comparable data to assess potential interspecific differences among pollinators, in this study, we adapted the standardized acute contact test for honey bees [56] to expose individual flies to a range of insecticide doses, build dose–response curves, and calculate LD_50_ values. We then followed a species sensitivity distribution curve (SSD) approach to compare the sensitivity of the three dipterans tested to that of other pollinators, including honey bees. This distribution statistically describes the variation among a set of species to a particular chemical or mixture, and attributes a fifth percentile hazard concentration/dose (HC5/HD5) that ensures a proper level of protection [60]. The SSD approach is a probabilistic approach to establish adequate protection exposure levels for most or all species when limited single-species toxicity data are available [61]. Based on previous studies showing large variability in pesticide sensitivity among different bee species [31], we expected large variability among the three dipteran species and between bees and dipterans.

The population effects of pesticide exposure not only depend on the intrinsic sensitivity (i.e., mortality) of the target species but also on their ability to reproduce during sublethal exposure, which is constrained by life history traits. Under stress conditions, some species prioritize investment in physiological maintenance and survival at the expense of reproduction. On the other hand, other species, often called opportunistic, maintain high levels of investment in reproduction until they reach levels of lethal exposure [62]. The population effects of a toxicant can be measured by the sublethal sensitivity index (SSI), which is the ratio between the LD_50_ or LC_50_ (median lethal dose or concentration) and the NOEL (no observed effect level) for reproduction [62]. For a given LD_50_, population effects will manifest earlier (at lower doses) in species with high SSI, that is, in species prioritizing energetic investment in somatic maintenance, including stress resistance and immunity [63]. Measuring reproductive output in the laboratory is not feasible for most bee species, which require flying space and large amounts of pollen and nectar resources [64]. However, by providing adequate oviposition substrates, fecundity (number of eggs laid) can be reasonably assessed in many dipterans under laboratory conditions. In this study, we assessed the effects of dosage on fecundity (number of eggs laid) and used the results to calculate the SSI for the three species.

The objectives of our study were: (1) to measure the contact toxicity of imidacloprid in three dipteran species using a methodology comparable to that used for bees; (2) to assess interspecific variation in sensitivity to imidacloprid using a sensitivity distribution curve approach; and (3) to assess the sublethal effects of imidacloprid exposure on fecundity and assess the vulnerability of the three species using the SSI approach.

## 2. Materials and Methods

### 2.1. Species, Populations, and Test Conditions

A population of *E. larvarum* was maintained at the Department of Agricultural and Food Sciences (DISTAL—University of Bologna, Italy) following the standard rearing procedures described by Dindo et al. [65]. *Galleria mellonella* L. (Lepidoptera: Pyralidae) larvae, reared at 30 ± 1 °C, 65 ± 5% relative humidity, and complete darkness, were used as a factitious host [66]. Mature *E. larvarum* puparia weighing 35–55 mg were selected for the experiment. Newly emerged individuals were kept in Plexiglas cages (40 cm × 30 cm × 30 cm) in a 1:1 sex ratio and left to mate for 3 days. Mated females were individually exposed to imidacloprid. Following exposure, six groups of five females of the same treatment group were transferred to Plexiglas cages (20 cm × 20 cm × 20 cm) within each of the eight treatments.

*Sphaerophoria rueppellii* were obtained from a population reared in Belgium (Spharophoria-System, Biobest N.V.) and shipped as pupae to DISTAL in February–April 2021. Upon arrival, pupae were introduced to standard mesh cages (60 cm × 40 cm × 40 cm) and transferred to a 26 °C chamber for incubation. Upon emergence, we let adults (1:1 sex ratio) mate for 2 days. Following the procedure previously described and after the exposure to imidacloprid, females were individually transferred to cages (V = 150 cc) with the lid perforated for air circulation and maintained there until the end of the test.

*Eristalinus aeneus* pupae were obtained from a population reared in Spain (Goldfly^®^, Polyfly S.L., Spain) and shipped to DISTAL in November 2021 and January 2022. Pupae were incubated as described above. Emerged individuals were kept in standard mesh cages (60 cm × 40 cm × 40 cm) for 5 days in a 1:1 sex ratio. Mated females were treated as described for *S. rueppellii*.

All flies were kept under light:dark conditions (16:8 h) in a climatic chamber at 26 ± 1 °C and 70–80% relative humidity during emergence and mating and for the duration of the test. Upon emergence, all fly species were fed *ad libitum*, with honey bee pollen pellets from organic beekeeping (Bona Mel^®^, Spain and sugar cubes. Pollen was supplied to promote ovary development [7]. Distilled water was provided in plastic 50 mL drinking straws (*E. larvarum*) and in 1 mL Eppendorf tubes with the tip cut off and plugged with dampened cotton (*S. rueppellii* and *E. aeneus*). Water was refilled as needed. Initial sample sizes were ca. 30 females per species and dose. Each female represented a replicate in the statistical analyses. A randomly selected group of mated females of each species (n = 26–55) were weighted to obtain an average measure of fresh body weight.

### 2.2. Imidacloprid Solutions

We used the commercially available formulation Confidor^®^, CropScience S.r.l., Italy (imidacloprid 20% *w*/*v*). Following preliminary range-finding tests, we started by diluting Confidor^®^ in HPLC-grade acetone at the highest tested concentration for each species (1563 ng·µL^−1^ for *E. larvarum*, 16 ng·µL^−1^ for *S. rueppellii*, and 43,750 ng·µL^−1^ for *E. aeneus*) and then we applied serial dilutions to achieve the appropriate doses.

### 2.3. Experimental Design, Exposure, and Mortality Assessment

For the three fly species, two negative control groups were included: negative control (untreated) and solvent control (pure acetone). To obtain imidacloprid dose–response curves, we exposed flies to six/seven doses in a geometric series. Following the preliminary range-finding trials, we used a dilution factor of 2.5 (5 imidacloprid doses from 40 to 1563 ng insect^−1^) in *E. larvarum*, 2.5 (5 doses from 0.41 to 16 ng insect^−1^) in *S. rueppellii*, and 3 (6 doses from 540 to 43,750 ng insect^−1^) in *E. aeneus*. After meeting, adult female flies were individually transferred to petri dishes and anesthetized on ice blocks for 2–3 min at 4–5 °C, and then exposed to the imidacloprid doses by topical application with 1 µL per female. The dose was applied on the dorsum of the thorax between the neck and the wing base by using a Gilson^®^ Repetman automatic precision micropipette (Middleton, WI USA). Mated females of the solvent control were exposed to pure acetone. In the negative control, females were anesthetized as in the other groups, but the exposure phase was only simulated Mortality was recorded at 4, 24, 48, 72 and 96 h after exposure and compared with solvent control values. The number of replicates (single females) for the estimation of the dose–response curves was ca. 30 per dose.

### 2.4. Sublethal Effect Assessment: Reproduction (Oviposition Rate and Fecundity)

In the same live females used for the assessment of mortality, fecundity was evaluated 24 h after the application of the insecticide. Fecundity assessment was adjusted to the life history of each species. In *E. larvarum*, we counted the number of eggs laid on host larvae. Mated females in groups of five were introduced in Plexiglas cages (20 cm × 20 cm × 20 cm) with last instar *G. mellonella* larvae (three larvae per female) for 1 h [67]. After the oviposition period, the overall number of eggs counted on the host larvae was divided by the number of females in each cage to determine fecundity, expressed as the average number of eggs laid per female in 1 h. Contrary to the other two species, oviposition rate (proportion of females that laid eggs) in *E. larvarum* was assessed at the cage level (five females per cage) rather than at the individual level. In *S. rueppellii*, eggs laid by each individual female were counted daily until the end of the test (96 h). In each cage, we added two sprouts of pea (*Pisum sativum* L.) infested with aphids (*M. persicae*) and one non-infested sprout to provide a free surface for egg laying. Aphid populations were maintained on pea plants in a climate chamber at 20 ± 1 °C, 60–80% relative humidity and 16:8 h (light:dark) photoperiod. Sprout roots were wrapped in humid (distilled water) cotton and aluminum foil to keep the plant alive. Sprouts and aphids were substituted as needed. Eggs were counted daily and hoverfly larvae were removed as detected to avoid egg predation [68]. Fecundity of individual *E. aeneus* females was assessed daily for 7 days. To promote egg laying, we provided a substrate of decaying soaked oat grains [69,70] for each individual female. The substrate was prepared with organic oat and distilled water (200 g per 175 mL) and left for 24 h at room temperature in complete darkness. Each female was provided with ca. 9.3 g of substrate, which was renewed every two days to avoid mold formation. Fecundity in *S. rueppellii* and *E. aeneus* was expressed as the daily number of eggs laid per female. Simple sizes (N) were 16–28 for *S. rueppellii*, 6–17 (groups of 5 flies) for *E. larvarum*, and 21–29 for *E. aeneus.*

### 2.5. Ecotoxicological Data from Literature

Median lethal doses (LD_50_; expressed in ng/insect and in µg/g of insect) of imidacloprid for several bee and beetle species were obtained from the literature (see Supplementary Materials, Table S1). Unfortunately, we could not find data for other pollinator groups (butterflies and wasps). To increase the number of species available for the analysis, LD_50_ values from both active ingredient and commercial products were included. Although the presence of co-formulants may influence the LD_50_ values [71,72], inspection of the data gathered did not show any consistent trend when both LD_50_ estimates based on formulated products and on the active ingredient were available for the same species (Table S1). LD_50_ active ingredient estimates for *Apis mellifera* (n = 6) show a high level of variability, indicating that other factors such as population origin, test conditions and laboratory procedures may also influence the results. Where more than one value per species was available, we used the mean to build the SSD curve.

### 2.6. Statistical Analysis

Dose–response models were fitted to mortality data at the different exposure doses for each species using the *drc* package [73] in R ver. 4.1.2 [74]. Mortality was corrected with Abbott’s formula [75] using the untreated control as a reference. Differences in mortality between the untreated and solvent control were analyzed with chi-squared tests, and minimal detectable differences (MDDs) were calculated with the *propTestMdd* function of the *EnvStats* package (considering a 0.80 associated power and a 0.05 *p*-value). The *mselect* function of *drc* was used to determine which model function was most appropriate based on the Akaike information criterion (AIC). Due to the non-monotonic response of *E. aeneus*, in this species, the dose–response curve was divided into the two ascending parts [76]. Then, the median lethal dose (LD_50_) values at 48 h were calculated with the *ED* function of the *drc* package. Weight-normalized LD_50_ values were calculated based on the mean fresh weight of each species (Figure S1).

The species sensitivity distribution (SSD) was fitted to a log-normal dataset with the values of 48 h LD_50_ using the package *ssdtools* [77]. From the resulting curve, we obtained the 5% (HD5, as the lower limit of the distribution) hazardous dose, and calculated the 95% confidence intervals (CIs, 1000 interactions). Using the approach of Arena and Sgolastra [28], we calculated the sensitivity ratio (R) between *A. mellifera* and the other pollinator species:

$$\left(R=\frac{{LD}_{50}\;A.\;mellifera}{{LD}_{50}\;pollinator}\right)$$

The reproduction endpoints were assessed only in individuals that survived at least 24 h after insecticide exposure. For *E. larvarum*, we assessed the number of eggs laid during 1 h. For *S. rueppellii* and *E. aeneus*, eggs were counted daily for 4 and 7 days, respectively. Firstly, we verified for both oviposition and fecundity that there were no significant differences between the two controls (untreated and solvent control). We use chi-squared and Mann–Whitney U tests for oviposition and reproduction, respectively. Minimal detectable differences (MDDs) on parametric tests were assessed as explained above. When no significant differences were found, solvent controls were used in the comparison with treated groups [78]. Then, the effect of treatment on oviposition rate (proportion of females that laid eggs) was examined with generalized linear models (GLMs) by fitting a binomial error distribution using the function *glm*. Gaussian error distribution was used to detect differences among treatments in fecundity (number of eggs laid per female). Data were log-transformed prior to analysis to achieve normal error distribution. All results are reported with a significance level of 5%.

The sublethal sensitivity index (SSI) is the ratio between the median lethal dose (LD_50_) and the no-observed-effect level (NOEL) for specific endpoints [62]. However, NOEL is highly dependent on sample size and dose selection [79]. For this reason, the lower confidence bound of the benchmark dose (BMDL) (i.e., the estimated lowest dose that produces an adverse response compared to the negative control) is often used instead of NOEL [79]. The BMD is the dose, derived from the estimated dose–response curve, associated with a given BMR (benchmark response, also known as critical effect size). The BMR was set at 10%, as recommended for quantal data analysis. In our study, BMDL values were estimated with the US EPA’s Benchmark Dose Software (BMDS 3.3.2 online version) [80] using the number of females that laid eggs (oviposition rate) for each treatment, and SSI was calculated for each species. High SSI values (>1) indicate that reproduction is a sensitive endpoint because it is inhibited at doses much lower than the LD_50_. Low SSI values (<1) indicate that the organism maintains its investment in reproduction until death.

## 3. Results

### 3.1. Species Sensitivity Distribution: Mortality

Mortality of the untreated control at 48 h, used to correct mortality of the other treatments, was 17% in *E. larvarum,* 0% in *S. rueppellii*, and 21% in *E. aeneus*. No significant differences were observed between the untreated and solvent control groups (*E. larvarum*: χ^2^ = 0.15, d.f. = 1, *p* = 0.70, MDD: 38%; *S. rueppellii*: χ^2^ = 1.48, d.f. = 1, *p* = 0.22, MDD: 29%; *E. aeneus*: χ^2^ = 0.083, d.f. = 1, *p* = 0.78, MDD: 36%). Imidacloprid dose–response curves varied substantially between species and the LD_50_ at 48 h increased in the following order: *S. rueppellii* < *E. larvarum* < *E. aeneus* (Table 1). This sensitivity ranking was maintained when LD_50_ values were corrected by the fresh body weight of each species (LD_50_ expressed in µg divided the average body weight expressed in grams). The dose–response curve of *E. aeneus* was non-monotonic and we could only calculate the LD_50_ associated with the first ascending part of the curve. Dose–response model parameters and LD_50_ values of the three dipteran species are reported in Table 1 and plots in the Supplementary Material (Figure S2).

**Figure 1:**
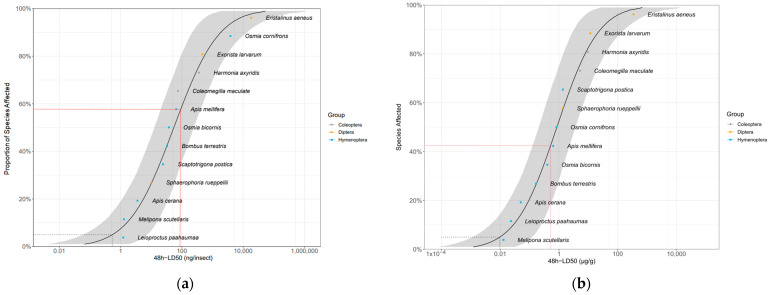
Distribution of sensitivity to imidacloprid calculated from 48 h LD_50_ values of three fly, eight bee and two beetle species, not accounting (**a**) and accounting (**b**) for fresh body weight. The gray area shows the parametric 95% CI (1000 interactions). The red dashed line indicates the LD_50_ for *Apis mellifera*. The gray dashed line indicates the HD5 hazardous dose (the dose protecting 95% of the species). LD_50_ values for bee and beetle species were obtained from the literature [81,82,83,84,85,86,87,88,89]. Values for *A. mellifera*, *Bombus terrestris*, *Osmia bicornis* and *Osmia cornifrons* are means from different studies [27,33,76,84,89,90,91,92,93,94,95,96] (Table S1).

We fitted the 48 h LD_50_ of our three target species and that of eight bee and two beetle species (obtained from the literature) into the SSD curve. The resulting HD5s (5% hazardous doses) were 0.615 ng/insect (95% CI [0.087; 7.416]) and 0.0105 µg/g of insect body weight (95% CI [0.0016; 0.118]) (Figure 1). The SSD curve shows that the sensitivity of *A. mellifera* is intermediate. That is, 59% (41% when body weight is accounted for) of the species are not protected by the honey bee LD_50_. Based on sensitivity ratio (R), the 10-fold safety factor recommended by the EFSA [27] to extrapolate the sensitivity of *A. mellifera* to other pollinators, was protective for 75% of the tested species, including the three dipterans, but not for three bee species (*Apis cerana* Fab., *Melipona scutellaris* Latreille and *Leioproctus paahaumaa* Donovan; Table S2).

### 3.2. Sublethal Effects: Reproduction (Oviposition Rate and Fecundity)

**Figure 2:**
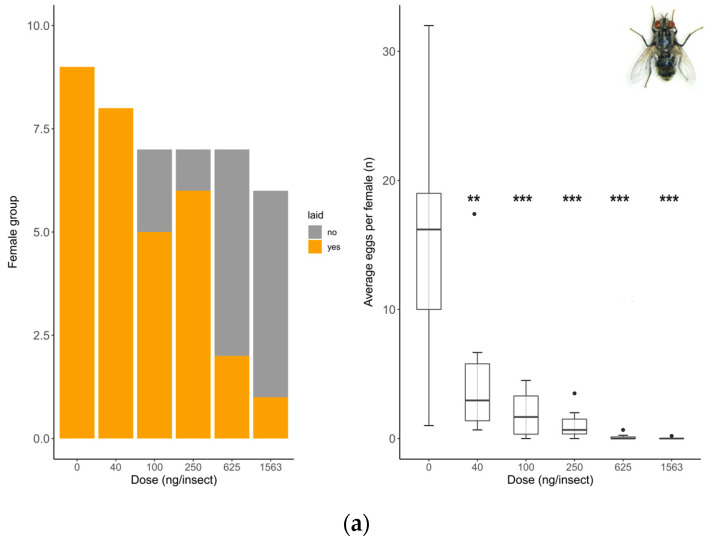

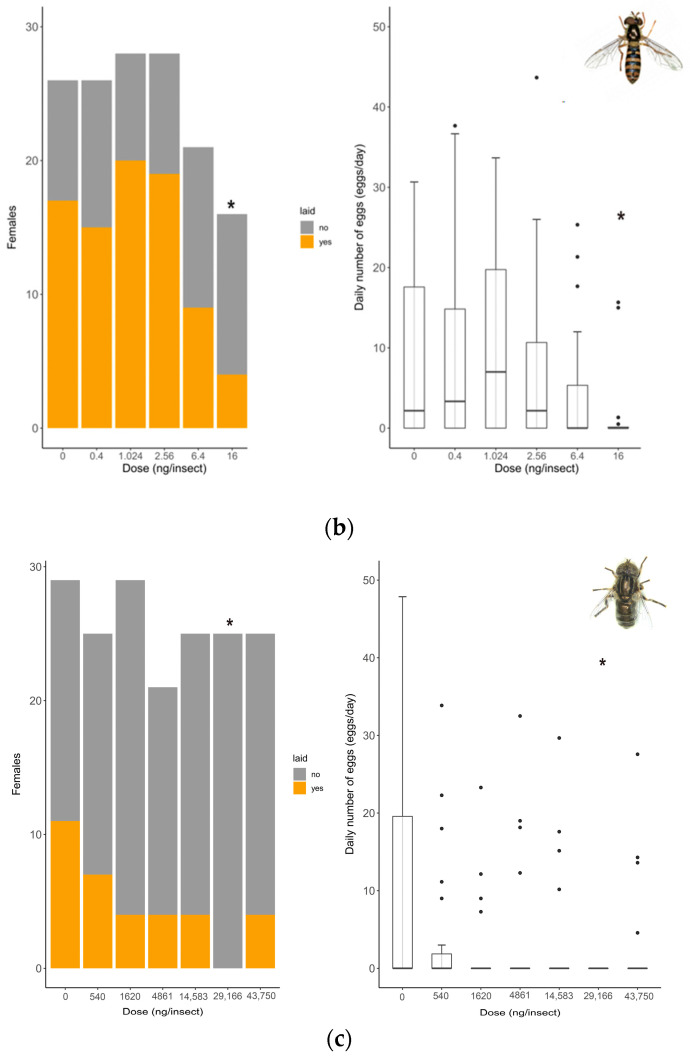
Reproduction comparison between control and treated females at different doses of Confidor^®^ (a.i. imidacloprid) in three dipteran species: *Exorista larvarum* (**a**), *Sphaerophoria rueppellii* (**b**), and *Eristalinus aeneus* (**c**). Bar plots indicate oviposition (the number of mated females that laid eggs), and box plots indicate the fecundity rate per female (*E. larvarum* number of eggs per female assessed during 1 h; *S. rueppellii* number of eggs per day per female assessed during 72 h; *E. aeneus* number of eggs per day per female assessed during 168 h. The line in the center of each box is the median, and the box spans the interquartile range (IQR), containing 50% of the data. Whiskers extend to the minimum and maximum values within 1.5 times the IQR. Outliers are also indicated. Bar plots and box plots with asterisks are significantly different from the control (* *p* <0.05; ** *p* < 0.01, *** *p* < 0.001). Simple size: *S. rueppellii* (N = 16–28), *E. larvarum* (N = 6–17 †), and *E. aeneus* (N = 21–29). † Groups of 5 flies per cage.

Oviposition rate (number of females that laid eggs) showed no differences between untreated and solvent control females in any of the species tested (*S. rueppellii*: χ^2^ = 3.4; d.f. = 1, *p* = 0.064, MDD: 37%; *E. aeneus*: χ^2^ = 0.42, d.f. = 1, *p* = 0.518, MDD: 39%). Analyses for *E. larvarum* were not conducted because the data were identical in both treatments. Fecundity (number of eggs laid per female) did not differ between untreated and solvent control females in any of the species tested either (*E. larvarum*: U = 43.0, d.f. = 1, *p* = 0.86; *S. rueppellii*: U = 276.5, d.f. = 1, *p* = 0.090; *E. aeneus*: U = 503.5, d.f. = 1, *p* = 0.214). For these reasons, only the solvent control was included in subsequent analyses. In *E. larvarum*, no differences were observed between treatments in oviposition (GLM: χ^2^ = 9.35 d.f. = 5, *p* = 0.090; Figure 2a). However, average eggs per female differed across treatments (GLM: χ^2^ = 122.34, d.f. = 5, *p* < 0.001; Figure 2a). All doses were significantly different from the control group (Figure 2a). In *S. rueppellii*, differences were observed among treatments in both oviposition (GLM: χ^2^ = 13.04, d.f. = 5, *p* = 0.023; Figure 2b) and fecundity (GLM: χ^2^ = 12.85, d.f. = 5, *p* = 0.025; Figure 2b). At the highest dose (i.e., 16 ng/insect) both endpoints were reduced compared to the control (Figure 2b). In *E. aeneus*, differences between treatments in oviposition were detected (GLM, χ^2^ = 12.68, d.f. = 6, *p* = 0.048, Figure 2c). The proportion of females that laid eggs was reduced both at 1620 and 29,166 ng/insects respect to the control (Figure 2c). In this species, fecundity was also different between treatments (GLM: χ^2^ = 18.17, d.f. = 6, *p* = 0.005; Figure 2c). All doses, with the exception of 540 ng/insect, were different from the control (Figure 2c). The BMDL calculated on oviposition (proportion of females laying eggs) for the three target species are shown in Table 2. All SSIs were > 1, indicating that oviposition was inhibited by exposure to the insecticide (Table 3).

## 4. Discussion

Most ecotoxicological studies on pollinators focus on the western honey bee, *A. mellifera*, and to a lesser extent bumblebees and solitary bees. Information on the sensitivity of other pollinator groups is mostly lacking, and when available, results are often difficult to compare with results of bee studies due to the use of different methodologies (i.e., oral bioassay [41], test on larvae [43]). Here, we provide relevant ecotoxicological endpoints for three dipteran pollinator species covering a range of life history traits and larval feeding habits, including a parasitoid, a predator, and a saprophagous species. As far as we know, this is the first study testing acute contact toxicity in dipteran pollinators using a risk assessment standard methodology. This is important because environmental risk assessment for pollinators is increasingly shifting towards a system-based approach integrating multiple species [40].

Among the three species tested, the aphidophagous hoverfly *S. rueppellii* exhibited the highest sensitivity to imidacloprid. High sensitivity of this species to this neonicotinoid was also reported in a previous study [97], in which adults were fed honeydew laced with imidacloprid at concentrations (15.6 ng/mL) detected in treated plants. On the other hand, the saprophagous hoverfly *E. aeneus* was by far the least sensitive species. This result is in line with other studies that observed a relatively high tolerance of another Eristalini, *Eristalis tenax* (L.), to imidacloprid and another neonicotinoid, thiamethoxam [41,43]. Interestingly, we obtained a non-monotonic dose response in *E. aeneus*. At intermediate doses, the toxicity dropped and started to increase again at higher doses. A similar biphasic hormetic dose–response pattern has been previously documented in several insects following exposure to neonicotinoids, including imidacloprid [76,98,99,100,101]. Hormetic response emerges when organisms mitigate stressor effects at low levels, often at the expense of other physiological processes. However, this balance becomes challenging to sustain at higher doses [102,103]. Numerous specific biochemical or molecular pathways could potentially contribute to hormesis, such as those associated with detoxification enzyme networks, oxidative stress response, and hormone signaling cascades [104,105,106,107]. Nonetheless, the precise reasons for the non-linear variation in neonicotinoid toxicity remain unclear and should be further explored.

The SSD curve shows a wide range of sensitivity to imidacloprid, which can be explained by differences in binding properties of the nicotinic acetylcholine receptors among species [108,109,110]. Interspecific variation in pesticide sensitivity is often phylogenetically constrained [111] and can also be partially explained by differences in body size [31,89] and other biological traits (e.g., hemolymph pH, lipid content, cuticle thickness). Especially when accounting for body weight, Coleoptera and Diptera species appear to be more tolerant to imidacloprid than bees. In this regard, toxicogenomics has strong potential to elucidate the molecular mechanisms evolved by different phylogenetic groups to respond to different toxicants. *Sphaerophoria rueppellii*, the most sensitive non-bee species in our SSD analysis, has a greater number of genes in various detoxification families, such as GSTs and CYP450s, in comparison with bees [112]. Some studies indicate that this expansion of detoxification genes in hoverflies may have evolved as an adaptation to exposure to environments with highly heterogeneous chemical backgrounds [112,113,114,115].

Our findings have important implications for environmental risk assessment. Bee pesticide risk assessment schemes rely almost exclusively on the western honey bee, *A. mellifera*, even though various studies have shown that this species is often less sensitive to pesticides than other bees [31,116]. For this reason, attempts to include other bee species in risk assessment schemes [27] have recently resulted in a roadmap towards a more holistic environmental risk assessment inclusive of all insect pollinator groups [40]. Species sensitivity distribution (SSD) curves are a useful approach to obtain estimates of pesticide sensitivity across species and to predict the proportion of potentially affected species at different concentrations using a limited set of ecotoxicity data [117]. Our results show that 60% of the species for which data are available, including the hoverfly S. *rueppellii*, fall below the honey bee LD_50_ threshold. When accounting for body weight, the level of protection derived from the honey bee LD_50_ increases, but still 40% of the species, all of them bees, remain unprotected. SSD curves can also be used to establish dose/concentration thresholds that would protect the 95th percentile of the species (HD5). In our study, the HD5 was approximately 100-fold lower than the LD_50_ of the honey bee. This value is 10 times higher than the safety factor of 10 recommended by the European Food Safety Authority [27] to estimate the sensitivity of untested bee species using honey bee data. Three (*A. cerana*, *M. scutellaris* and *L. paahaumaa*) of the 13 tested species in our dataset would have LD_50_ values lower than the honey bee LD_50_ even when applying a correction factor of 10. This outcome is in line with other studies emphasizing the need to apply safety factors higher than 10 to protect non-*Apis* bees [37,38]. The potential of the SSD approach is limited by the number and phylogenetic composition of species for which reliable LD_50_ values are available. Our study underscores the need to include a range of model species in pollinator and non-target arthropod risk assessment, including underrepresented insect orders such as Diptera and Lepidoptera [40]. Importantly, similar experimental procedures should be used across taxa so as to reduce the potential impact of confounding factors in the assessment of pesticide sensitivity. This is also important at the species level, as evidenced by the high variability found in the literature for honey bee LD_50_ estimates. To overcome this potential shortcoming, it is important to include a toxic reference in the analysis. Toxic references in risk assessment are used as a mortality reference and to validate the response of the test system by ensuring exposure consistency and the sensitivity of the test. Dimethoate LD_50_ is used as a toxic reference in bee risk assessment schemes, but no information is available for other pollinator species. Therefore, in our study, the results of a positive control could not be compared to a reference standard. Future studies should address the establishment of toxic reference values for non-bee pollinators.

Regulatory bodies also need repeatable methods to generate reliable data on intrinsic sensitivity. However, the ultimate impact of a pesticide will also depend on population resilience [118]. The latter may be defined as the capacity of the population to recover following disturbance, which is directly linked to oviposition and fecundity. Declines in these parameters can be considered early warning signals (EWSs) before population collapse. We assessed the effect of imidacloprid exposure on oviposition and fecundity in the three dipteran species. Again, *S. rueppellii* was the most sensitive species, followed by *E. larvarum* and *E. aeneus*. The lower confidence bound of the benchmark dose (BMDL) for oviposition in this species was 28 times lower than that of *E. larvarum* and 210 times lower than that of *E. aeneus*. Different species show different strategies and/or levels of adaptive plasticity to allocate resources between individual maintenance (survival) and reproduction. The sublethal sensitivity index (SSI) is a useful approach to predict the ecological effects of a pesticide, where a high SSI value indicates a high level of vulnerability. In agreement with results obtained in other invertebrate groups [62], SSI values for the three dipterans analyzed in our study were higher than 1, indicating that oviposition is a sensitive endpoint in these species, and therefore represents a useful EWS. The sublethal (BMDL) doses were 6 to 50 times lower than the lethal (LD_50_) doses, indicating that these species reduce the investment in oviposition in the presence of a pesticide stressor.

## 5. Conclusions

In conclusion, our study provides new information on the sensitivity of dipteran species and demonstrates that it is possible to apply standardized bee ecotoxicological procedures to other pollinator groups. Future studies should explore the mechanisms that cause non-monotonic dose–response relationships and create standardized toxicity testing techniques for dipteran pollinators. The integration of our results into SSD curves represents a first step towards an advanced environmental risk assessment, covering an expanded pool of species and including non-bee pollinators. The possibility of measuring oviposition and fecundity (and fertility in future studies) in some dipteran species under laboratory conditions, together with the use of SSI, widens our ability to predict the impact of pesticides at the population level and provides a crucial first step for the integration of population dynamics in simulation models for environmental risk assessment [119].

## Figures and Tables

**Table 1:** LD_50_ values at 48 h (in ng/insect and µg/g of insect body weight) and dose–response model parameters for three dipteran species following acute topical exposure to Confidor^®^ (a.i. imidacloprid). * Values for *Eristalinus aeneus* were split for the two ascending parts of the dose–effect curve (LD_50_ could only be calculated considering the first ascending part of the curve. The upper doses were not included in the model).

Species	n	Model	Slope	Log-Likelihood	Residual Standard Error (df)	AIC	*p*-Value	48 h LD_50_	95% CI	48 h LD_50_	95% CI
								(ng/Insect)		(µg/g Insect)	
*Exorista larvarum*	233	Log-logistic	−0.765	−14.87	4.89 (3)	37.75	0.0087	467.46	302.28–632.65	11.66	7.54–15.79
*Sphaerophoria rueppellii*	205	Log-logistic	−1.668	−19.35	5.11 (3)	46.85	0.012	10.23	7.81–12.65	1.35	0.83–1.86
Eristalinus aeneus *	193	Log-logistic	−1.062	−9.86	2.75 (2)	27.74	0.036	18,176.20	8005.6–28,346.9	344.77	151.85–537.69

**Table 2:** BMDL (lower confidence bound of the benchmark dose), BMD (benchmark dose) and BMDU (upper confidence bound of the benchmark dose) that affected oviposition rate (number of females that laid eggs) in ng/insect in three dipteran species. A BMR (benchmark response) of 10% change after acute topical exposure to Confidor^®^ (a.i. imidacloprid) was considered. Best-fit models and their related parameters for the three dipteran species are provided; n = sample size of females that survived >24 h after exposure.

Species	n	Model	BMDL	BMD	BMDU	*p*-Value	AIC
			(ng/Insect)	(ng/Insect)	(ng/Insect)		
*Exorista larvarum*	178	Multistage 1°	47.08	75.11	127.12	0.639	33.535
*Sphaerophoria rueppellii*	174	Logistic	1.70	2.50	4.69	0.649	190.797
*Eristalinus aeneus*	209	Log-Logistic	356.79	1080.91	8498.47	0.178	165.298

**Table 3:** Comparison of lethal (LD_50_, median lethal dose at 48 h) and sublethal (BMDL, lower confidence bound of the benchmark dose that affects oviposition) doses after acute topical exposure to Confidor^®^ (a.i. imidacloprid) in three dipteran species. SSI = sublethal sensitivity index (LD50/BMDL).

Species	LD_50_	BMDL	SSI
	(ng/Insect)	(ng/Insect)	
*Exorista larvarum*	467.46	47.08	9.93
*Sphaerophoria rueppellii*	10.23	1.70	6.01
*Eristalinus aeneus*	18,176.20	356.79	50.94

## Data Availability

Data will be made available on request.

## Supplementary Materials

The following supporting information can be downloaded at https://www.mdpi.com/article/10.3390/insects15050317/s1. Figure S1: Boxplots of fresh weight for the three fly species.; Table S1: Toxicity data used to build the species sensitivity distribution (SSD) curve. LD_50_ values: median lethal dose of imidacloprid at 48 h. Where no weight standardization was performed in the original study, we indicate the reference for the mean weight used to transform the data.; Table S2: Species sensitivity ratio (R). Values in bold exceed the range of 10-fold safety factor from the endpoint of *A. mellifera*, recommended by the EFSA (2013); Figure S2: Fitted dose–response curves in (A) *Exorista larvarum* (L.), (B) *Sphaerophoria rueppellii* (Wiedemann) (Diptera: Syrphidae) and (C) *Eristalinus aeneus* (Scopoli) (Diptera: Syrphidae). Ref. [120] is cited in Supplementary Materials.
