# Supplementary material for: Different Sensitivity of Flower-Visiting Diptera to a Neonicotinoid Insecticide: Expanding the Base for a Multiple-Species Risk Assessment Approach
Source: Insects. 2024 Apr 29;15(5):317. doi: 10.3390/insects15050317 (PMC11122312; doi:10.3390/insects15050317)
Supplement: Supplementary file 1 [file insects-15-00317-s001.zip › insects-2958430-supplementary.pdf]

Supplementary material

Sensitivity of flower-visiting Diptera to a neonicotinoid insecticide: expanding the base for a multiple-species risk assessment approach

Cátia Ariana Henriques Martins[1], Celeste Azpiazu[2,3], Jordi Bosch[2], Giovanni Burgio[1], Maria Luisa Dindo[1], Santolo Francati[1], Daniele Sommaggio[1], Fabio Sgolastra[1*]

[1]*University of Bologna – Department of Agricultural and Food Science*
[2]*CREAF, Universitat Autònoma de Barcelona, E08193 Bellaterra, Spain*
[3]*Universidad Politécnica de Madrid, 28040 Madrid, Spain*

*Corresponding author: fabio.sgolastra2@unibo.it

Figure S1. Boxplots of fresh weight for the three fly species.

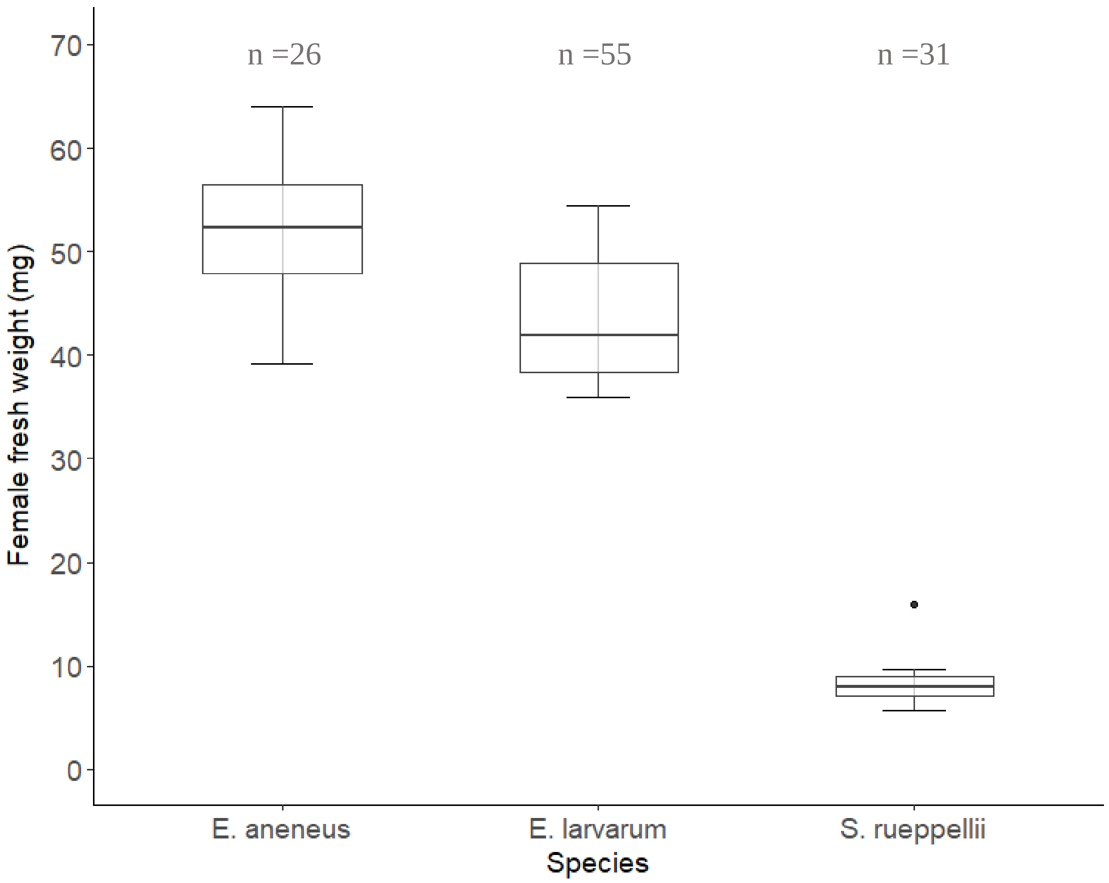

Table S1. Toxicity data used to build the Sensitivity Species Distribution (SSD) curve. LD_{50} values: median lethal dose of imidacloprid at 48h. Where no weight standardization was performed in the original study, we indicate the reference for the mean weight used to transform the data.

Species	Family	Mean fresh weight (mg)	Contact LD50 (ng/insect)	Contact LD50 (µg/g insect)	Formulation[1]	Reference
Flies (Diptera)						
Exorista larvarum	Tachinidae	40.1	467.5	11.7	c.f	This study
Sphaerophoria rueppellii	Syrphidae	7.9	10.2	1.35	c.f	This study
Eristalinus aeneus	Syrphidae	52.7	18176.2	344.8	c.f	This study
Bees (Hymenoptera)						
Apis mellifera	Apidae	100a	6.7	0.07	a.i.	[1]
Apis mellifera	Apidae	100a	24.3	0.24	a.i.	[1]
Apis mellifera	Apidae	100a	81	0.81	a.i.	[2]
Apis mellifera	Apidae	100a	42	0.42	c.f.	[2]
Apis mellifera	Apidae	100a	245	2.45	a.i.	[3]
Apis mellifera	Apidae	112	234	2.09	a.i.	[4]
Apis mellifera	Apidae	100a	150	1.5	c.f.	[5]
Apis mellifera	Apidae	100a	60	0.6	a.i.	[6]
Apis cerana	Apidae	75b	3.6	0.05	a.i.	[7]
Bombus terrestris	Apidae	200c	14	0.07	a.i.	[8]
Bombus terrestris	Apidae	200c	77	0.39	c.f.	[8]
Scaptotrigona postica	Apidae	18d	24.5	1.36	a.i.	[9]
Melipona scutellaris	Apidae	100e	1.29	0.013	a.i.	[10]
Leioproctus paahaumaa	Colletidae	52.4	1.21	0.0231	a.i.	[4]
Osmia bicornis	Megachilidae	94.6f	30	0.33	a.i.	[3]
Osmia bicornis	Megachilidae	94.6f	46	0.49	a.i.	[11]
Osmia cornifrons	Megachilidae	131g	3820	29.16	c.f.	[5]
Osmia cornifrons	Megachilidae	100-110	-	0.023	a.i.	[12]
Beetles (Coleoptera)						
Harmonia axyridis	Coccinellidae	37.5g	360	9.60	c.f.	[13]
Coleomegilla maculata	Coccinellidae	14.2g	74	5.21	c.f.	[14]

[1] a.i.: active ingredient, c.f.: commercial formulation

a. Mean weight from [15]
b. Mean weight from [16]
c. Mean weight from [17]
d. Mean weight from [18]
e. Attributed the same weight as honey bee *Apis mellifera* L.[19]
f. Mean weight from [3]
g. [20]

Table S2. Species sensitivity ratio (R). Values in bold exceed the range of 10-fold safety factor from the endpoint of *A. mellifera*, recommended by [2].

**Species**	**R (ng/insect)**	**R (µg/g insect)**
Exorista larvarum	0.14	0.05
Sphaerophoria rueppellii	6.27	0.47
Eristalinus aeneus	0.00	0.00
Apis cerana	**17.81**	**12.69**
Bombus terrestris	1.95	3.84
Scaptotrigona postica	2.62	0.47
Melipona scutellaris	**49.71**	**49.20**
Leioproctus paahaumaa	**52.99**	**27.48**
Osmia bicornis	1.73	1.58
Osmia cornifrons	0.02	0.78
Harmonia axyridis	0.18	0.07
Coleomegilla maculata	0.87	0.12

Figure S2. Fitted dose-response curves in (A) *Exorista larvarum* (L.), (B) *Sphaerophoria rueppellii* (Wiedemann) (Diptera: Syrphidae) and (C) *Eristalinus aeneus* (Scopoli) (Diptera: Syrphidae)

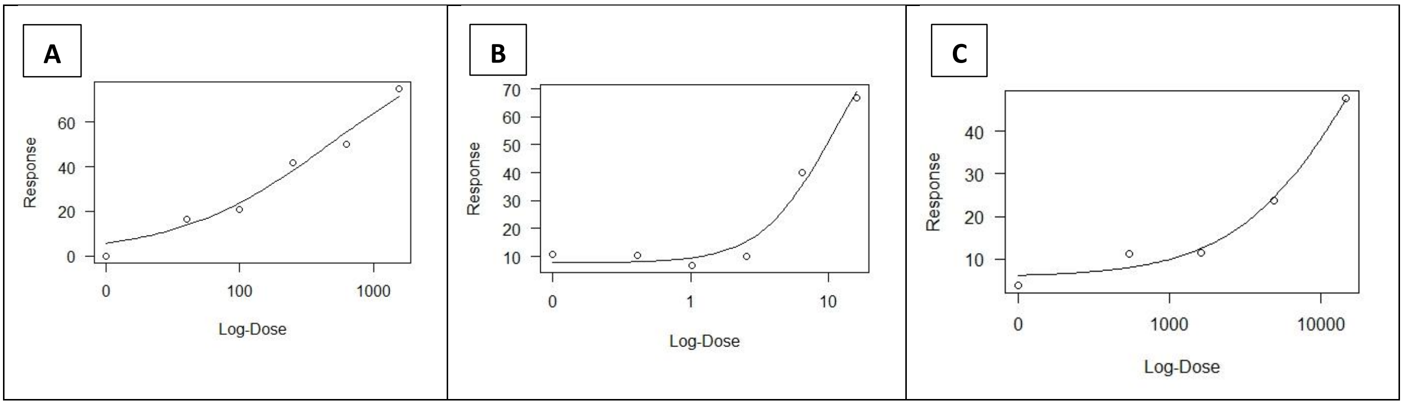

References

1. Suchail, S.; Guez, D.; Belzunces, L.P. Characteristics of Imidacloprid Toxicity in Two Apis Mellifera Subspecies. *Environ. Toxicol. Chem.* **2000**, *19*, 1901–1905, doi:10.1002/etc.5620190726.
2. EFSA Guidance on the Risk Assessment of Plant Protection Products on Bees (Apis Mellifera, Bombus Spp. and Solitary Bees). *EFSA J.* **2013**, *11*, 3295, doi:10.2903/j.efsa.2013.3295.
3. Uhl, P.; Awanbor, O.; Schulz, R.S.; Brühl, C.A. Osmia Bicornis Is Rarely an Adequate Regulatory Surrogate Species. Comparing Its Acute Sensitivity towards Multiple Insecticides with Regulatory Apis Mellifera Endpoints. *PLoS One* **2019**, *14*, e0201081, doi:10.1101/366237.
4. Kueh Tai, F.; Pattemore, D.E.; Jochym, M.; Beggs, J.R.; Northcott, G.L.; Mortensen, A.N. Honey Bee Toxicological Responses Do Not Accurately Predict Environmental Risk of Imidacloprid to a Solitary Ground-Nesting Bee Species. *Sci. Total Environ.* **2022**, *839*, 156398, doi:10.1016/j.scitotenv.2022.156398.
5. Biddinger, D.J.; Robertson, J.L.; Mullin, C.; Frazier, J.; Ashcraft, S.A.; Rajotte, E.G.; Joshi, N.K.; Vaughn, M. Comparative Toxicities and Synergism of Apple Orchard Pesticides to Apis Mellifera (L.) and Osmia Cornifrons (Radoszkowski). *PLoS One* **2013**, *8*, e72587, doi:10.1371/journal.pone.0072587.
6. ECOTOX Curated Toxicity Data Were Retrieved from the ECOTOXicology Knowledgebase. U.S. Environmental Protection Agency Available online: http:/www.epa.gov/ecotox/ (accessed on 1 November 2022).
7. Yasuda, M.; Sakamoto, Y.; Goka, K.; Nagamitsu, T.; Taki, H. Insecticide Susceptibility in Asian Honey Bees (Apis Cerana (Hymenoptera: Apidae)) and Implications for Wild Honey Bees in Asia. *J. Econ. Entomol.* **2017**, *110*, 447–452, doi:10.1093/jee/tox032.
8. Bortolotti, L.; Porrini, C.; Sbrenna, G. Effetti Dell'imidacloprid Nei Confronti Di Bombus Terrestris (L.). Prove Di Laboratorio. *Inf. Fitopatol.* **2002**, *3*, 66–71.
9. Soares, H.M.; Jacob, C.R.O.; Carvalho, S.M.; Nocelli, R.C.F.; Malaspina, O. Toxicity of Imidacloprid to the Stingless Bee Scaptotrigona Postica Latreille, 1807 (Hymenoptera: Apidae). *Bull. Environ. Contam. Toxicol.* **2015**, *94*, 675–680, doi:10.1007/s00128-015-1488-6.
10. da Costa, L.M.; Grella, T.C.; Barbosa, R.A.; Malaspina, O.; Nocelli, R.C.F. Determination of Acute Lethal Doses (LD50 and LC50) of Imidacloprid for the Native Bee Melipona Scutellaris Latreille, 1811 (Hymenoptera: Apidae). *Sociobiology* **2015**, *62*, doi:10.13102/sociobiology.v62i4.792.
11. Beadle, K.; Singh, K.S.; Troczka, B.J.; Randall, E.; Zaworra, M.; Zimmer, C.T.; Hayward, A.; Reid, R.; Kor, L.; Kohler, M.; et al. Genomic Insights into Neonicotinoid Sensitivity in the Solitary Bee Osmia Bicornis. *PLOS Genet.* **2019**, *15*, e1007903, doi:10.1371/journal.pgen.1007903.
12. Phan, N.T.; Joshi, N.K.; Rajotte, E.G.; López-Uribe, M.M.; Zhu, F.; Biddinger, D.J. A New Ingestion Bioassay Protocol for Assessing Pesticide Toxicity to the Adult Japanese Orchard Bee (Osmia Cornifrons). *Sci. Rep.* **2020**, *10*, 9517, doi:10.1038/s41598-020-66118-2.
13. Youn, Y.N.; Seo, M.J.; Shin, J.G.; Jang, C.; Yu, Y.M. Toxicity of Greenhouse Pesticides to Multicolored Asian Lady Beetles, Harmonia Axyridis (Coleoptera: Coccinellidae). *Biol. Control* **2003**, *28*, 164–170, doi:10.1016/S1049-9644(03)00098-7.
14. Lucas, É.; Giroux, S.; Demougeot, S.; Duchesne, R. -M.; Coderre, D. Compatibility of a Natural Enemy, Coleomegilla Maculata Lengi (Col., Coccinellidae) and Four Insecticides Used against the Colorado Potato Beetle (Col., Chrysomelidae). *J. Appl. Entomol.* **2004**, *128*, 233–239, doi:10.1111/j.1439-0418.2004.00843.x.
15. Suchail, S.; Debrauwer, L.; Belzunces, L.P. Metabolism of Imidacloprid in Apis Mellifera. *Pest Manag. Sci.* **2004**, *60*, 291–296, doi:10.1002/ps.772.
16. Thompson, H. Extrapolation of Acute Toxicity across Bee Species. *Integr. Environ. Assess. Manag.* **2016**, *12*, 622–626, doi:10.1002/ieam.1737.
17. Hagen, M.; Wikelski, M.; Kissling, W.D. Space Use of Bumblebees (Bombus Spp.) Revealed by Radio-Tracking. *PLoS One* **2011**, *6*, e19997, doi:10.1371/journal.pone.0019997.
18. Hartfelder, K.; Engels, W. Allometric and Multivariate Analysis of Sex and Caste Polymorphism in the Neotropical Stingless Bee,Scaptotrigona Postica. *Insectes Soc.* **1992**, *39*, 251–266, doi:10.1007/BF01323946.
19. Lourenço, C.T.; Carvalho, S.M.; Malaspina, O.; Nocelli, R.C.F. Oral Toxicity of Fipronil Insecticide Against the Stingless Bee Melipona Scutellaris (Latreille, 1811). *Bull. Environ. Contam. Toxicol.* **2012**, *89*, 921–924, doi:10.1007/s00128-012-0773-x.
20. Hätönen, M.; Kantner, C.; Lopez Losada, R.; Ludwig, N.; Benavent González, A.; Riedhammer, C.; Kunz, P.; Panico, S.C.; Laakkonen, E.; Parramon Dolcet, L.; et al. *European Arthropods and Their Role in Pollination: Scientific Report of Their Biodiversity, Ecology and Sensitivity to Biocides*; European Chemicals Agency: Helsinki, Finland, 2022; ISBN 978-92-9468-131-7.
